# Hydroelectric Dam May Impact the Microbiome of an Endangered Northern Map Turtle (*Graptemys geographica*) Population

**DOI:** 10.1007/s00284-026-04846-w

**Published:** 2026-03-28

**Authors:** Hailey A. M. Christoph, Steven J. A. Kimble

**Affiliations:** Department of Biological Sciences, Towson University, Towson, MD 21252 USA

## Abstract

Classified as state-endangered, Maryland’s population of Northern Map Turtles (NMTs) (*Graptemys geographica)* faces habitat alteration and fragmentation due to the Conowingo Hydroelectric Dam and other factors. Maryland’s NMTs also reside in an urbanized area with high levels of human activity, which leads to overall disturbance and pollution. The combined impacts of these stressors could be affecting the health of NMTs and may contribute to future decline. The microbiome has been explored as an indicator of organismal health in other species and can be used to inform wildlife management strategies. We collected cloacal (*n* = 35) and oral (*n* = 34) microbiome swabs from wild NMTs at four different sites along the Maryland portion of the Susquehanna River in 2022. Using 16 S rRNA sequencing, results suggested that the dam plays a significant role in determining cloacal and oral microbiome structure, with bacteria associated with pollution and disease being significantly more prevalent above the dam. Of note are *Acinetobacter spp.*, which is associated with heavy metal contamination, and *Aeromonas spp.*, which is associated with shell disease in map turtles. Despite the known dietary dimorphism of NMTs, we did not find significant differences in the microbiomes of males and females. The impact that dams have on microbiomes and wildlife is not well understood, and these results may help to enact more specific management strategies in these systems. Our findings will contribute to the conservation of an endangered freshwater turtle population while adding to the limited breadth of knowledge of reptilian health.

**Supplementary Information:**

The online version contains supplementary material available at 10.1007/s00284-026-04846-w.

## Introduction

Freshwater turtles account for 75% of all turtle species [1] and face many challenges associated with human disturbance of their habitats. Many of these populations experience significant habitat loss and fragmentation due to the construction of river disruptors such as locks and dams, which impairs their ability to find food and mates [2]. In turn, this may reduce survivorship and genetic diversity within those populations. Additionally, these species leave the water to nest on land, which, in developed areas, may cause them to experience high levels of road mortality and increased predation [3]. In addition to the threats outside of the water, freshwater turtles are often hit by boats, drowned in nets, or killed by fishhooks [3]. Freshwater turtles also have a high tendency to bioaccumulate pollutants because of their high trophic status and inclination to absorb or ingest contaminants in their environment [4]. Although freshwater turtle species face many threats and experiencing population declines, they remain relatively understudied compared to their marine counterparts.

One freshwater turtle species of interest is the Northern Map Turtle *(Graptemys geographica)*, which can be found in major rivers and lakes of central and eastern North America. Named for the map-like lines on the skin and carapace, this species is highly aquatic and typically only comes on land to bask or nest [10]. In riverine environments, they prefer well-oxygenated water with moderate flow and turbidity [10, 11]. Human-induced alterations to river flow, water quality, and habitat can threaten Northern Map Turtle biology, which likely makes them one of the most imperiled groups of turtles in the United States [11]. In Maryland, this species is listed as state-endangered due to having a small population size, limited dispersal, and significant habitat degradation [12]. The Maryland population resides exclusively on the Susquehanna River, which is the largest tributary of the Chesapeake Bay [13]. Maryland’s Northern Map Turtles face many potential threats, including the Conowingo Hydroelectric Dam, which bisects the population [12].

One potential tool that may be useful in understanding the effects of ecosystem disturbances on freshwater turtles is a microbiome analysis, which has been applied to the conservation of various species [5] by serving as a bioindicator of population health [6]. Several studies have already successfully utilized microbiomes to improve organismal health and survival. A study in artificially reared whooping crane chicks who received microbial transplants from healthy adults had reduced rates of disease and improved growth [5]. Use of natural substrates in captive salamanders showed that they retained a healthier microbiome in comparison to those with an artificial substrate [7], and skin inoculation with probiotics may decrease pathogenic fungal *Batrachochytrium dendrobatidis* (Bd) susceptibility in many amphibian species [8]. Use of these tools in turtle microbiome research remains sparse, despite over half of all species being listed as threatened or endangered [9].

Although they can reduce energy-related CO_2_ emissions and provide flood control, hydroelectric dams have been shown to be quite destructive to natural habitats and can negatively impact biodiversity [14], including host microbiomes [15]. The Conowingo Dam, completed in 1928, is the largest dam within the complex of dams on the Lower Susquehanna River [13] and forms the 3,600 ha Conowingo Reservoir [20]. In recent decades, it has shown a substantial increase in sediment load, trapping excess nitrogen, phosphorus, and other potential pollutants [21]. The reservoir is thought to be at or near capacity, allowing much of this sediment to spill during storms to the other side of the dam and into the Chesapeake Bay, which has negative impacts on Bay health [22]. The high sediment load above the dam means that turtles at those sites could be exposed to large amounts of these relatively high levels of nutrients and pollutants. However, since sediment does spill over to sites below the dam, especially in scour events [13], it is likely that turtles below the dam are also experiencing some level of contaminant exposure. Exposure to environmental contaminants may cause shell deformities in Northern Map Turtles, affecting their ability to swim and tolerate external stress [23]. Likewise, changes to river discharge and permanent flooding caused by the dam could reduce nesting availability and affect survivorship of nests and hatchlings [16]. Map turtles are also thought to be anoxia intolerant, so the poor water oxygen quality caused by the dam may influence their ability to overwinter [18, 19] due to decreased diving ability [17].

Changes to river flow from the dam have encouraged the establishment of invasive mollusks and shifted prey base for turtles on both sides of the Conowingo Dam [24, 25]. Previous dietary analyses in this population have shown that females above the dam primarily feed on zebra mussels (*Dreissena polymorpha*) [24], while those below feed on pleurocerid snails [25]. Males above the dam consume mystery snails (*Cipangopaludina japonica* and *C. chinensis*) and males below consume Asian clams (*Corbicula fluminea*) [25]. Considering the dietary sexual dimorphism of Northern Map Turtles, the biological and ecological consequences of these differences at a population level are unknown.

There is also an additional threat of human activity, as this is an urbanized area that is becoming increasingly more developed [12]. Construction of docks, residencies, and marinas along rivers were shown to be disruptive to nesting and basking sites in a similar map turtle species, which thus can impact nesting ability, predation, parasite removal and healthy egg development [26]. One of the known nesting sites of Northern Map Turtles in Maryland is Port Deposit, a small town below the dam that relies on tourism and recreation [12]. Recreational boating significantly increases the likelihood of injury to Northern Map Turtles, which can cause severe damage to shells, brain damage, and death [27]. These injuries can have delayed effects, such as increased infection and predation risk.

Maryland’s Northern Map Turtle population is separated by a physical barrier, the dam, and likely experiences various types of habitat disruptors upstream and downstream. Previous unpublished radio transmitter data of this population suggested that the turtles do not cross the dam, and typically move between areas on the same side. Since the microbiome can be heavily altered by stress, diet, habitat changes and pollutants, the microbiome is likely disrupted in Northern Map Turtles in different ways above and below the dam. This could include significant discrepancies in microbial evenness, abundance, diversity, and composition of the turtles living in these two areas. Various fish species in a dam-disrupted river in China showed higher diversity and abundance of bacteria involved in energy metabolism above the dam compared to below, likely due to the different demands of each habitat [15]. Combining habitat-mediated microbial changes with the exposure to contaminants, physical separation, and loss of food sources, these factors could have physiological and phenotypic implications and further the endangerment of Maryland’s Northern Map Turtles.

In this study, we aim to characterize the cloacal and oral microbiomes of Maryland’s Northern Map Turtles on both sides of the Conowingo Dam. We examine how these microbial communities differ upstream and downstream of the dam by identifying abundant bacterial taxa and comparing diversity between the two sides. We also address potential sex-related differences in the microbiome composition of this population. The goal of this study is to use these findings to identify areas or microbes of concern to enhance conservation efforts focused on Northern Map Turtles in Maryland.

## Materials and Methods

### Study Site

Northern Map Turtles were sampled at various locations along the Susquehanna River in Northeast Maryland, USA. The Susquehanna is the largest tributary of the Chesapeake Bay watershed, accounting for more than half of the flow of freshwater into this system [20]. The lower Susquehanna contains several dam reservoirs, all of which reduce sediment and nutrient transport into the Bay [20]. The largest of these is the Conowingo reservoir, a focal point of this study. We selected two sites above and below the Conowingo Dam to sample turtles. The two sites above the dam, Conowingo Creek and Broad Creek, are likely to contain higher amounts of excess sediment and poorer water quality compared to sites below the dam [20]. Prior water quality assessments of Conowingo Creek (~ 3 km upstream from the dam) showed higher levels of dissolved oxygen, excess nitrate, ammonium, and orthophosphate. Levels of *E.coli* have also been reported to be above the safety limit for humans at this location [28]. Broad Creek (~ 7 km upstream from the dam) is a large inland stream that is frequently used for recreation [29], but limited water quality data exists for this site. The two sites below the dam at the town of Port Deposit and at an unnamed nesting beach. The nesting beach is an isolated, relatively natural area where nest predation is high. The field station at Port Deposit (~ 8 km downstream from the dam) is located in the town limits between a city park and a marina. Turtles have historically nested in this area, despite the high likelihood of being disturbed due to human activity, poorer quality nest substrate, and increased recreational boating [30]. Turtles have historically moved between sites on the same side of the dam but do not cross to the other side, according to previous unpublished radio transmitter data.

**Fig. 1:**
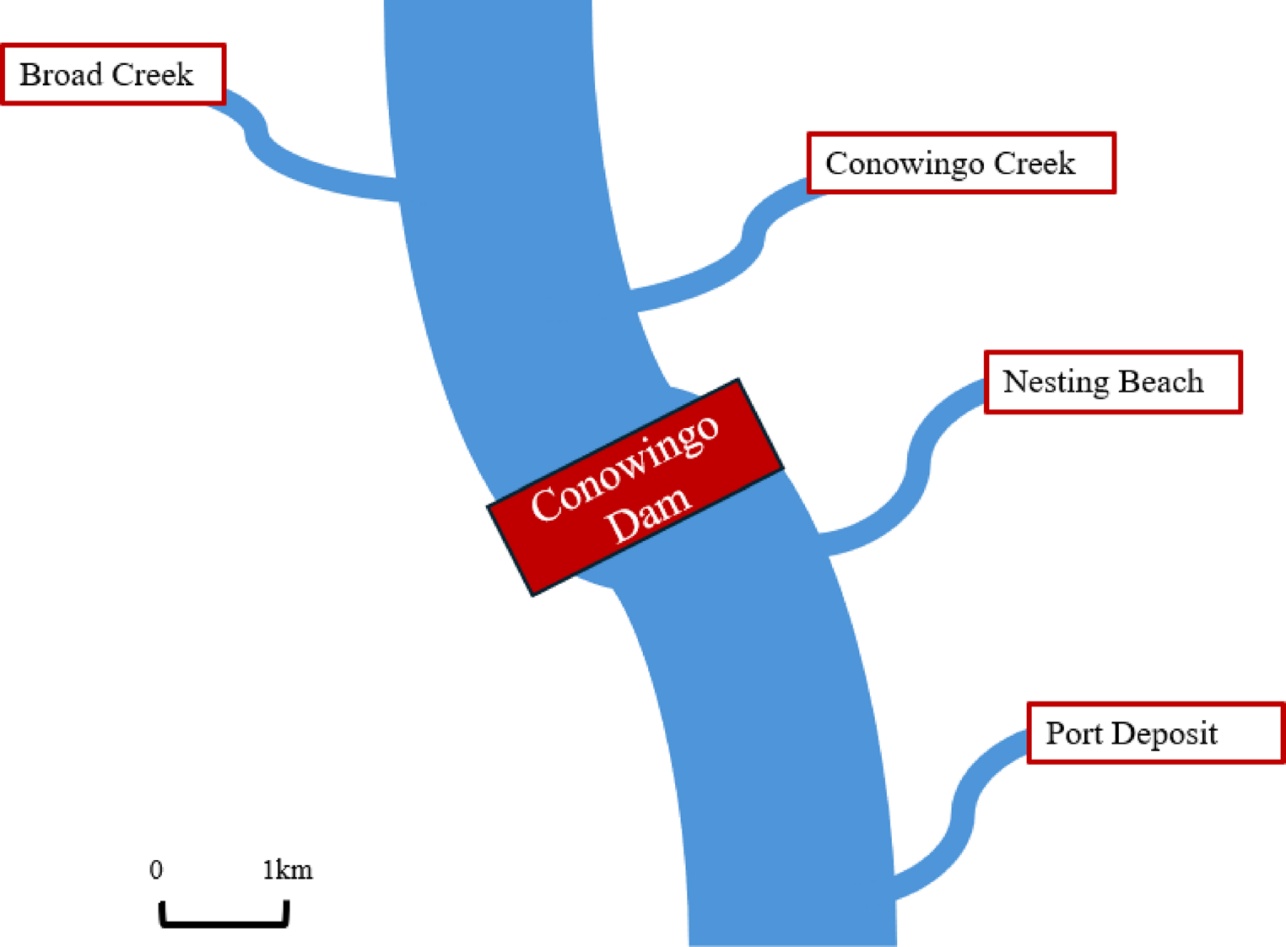
Map showing relative locations of sample sites along the Maryland portion of the Susquehanna River, USA

### Field Sample Collection

Northern Map Turtles were caught by hand, basking trap or hand net at the various sites in May-June 2022 [25]. Basking traps were nailed to natural logs where turtles were observed basking upon arrival to the sample site. When turtles were observed on logs that had traps on them, we approached in a kayak to get them into the trap below. Traps were removed at the end of the day. Once captured, individuals were transported to the field site in Port Deposit for further processing. Nitrile gloves were worn when handling the turtle to prevent contamination. While at the field site, turtles were placed in separate 40 L plastic bins during the processing and data collection step. Tools and bins were sanitized using 10% bleach between turtles to help prevent contamination and reduce potential disease spread [24]. Individuals were identified based on marginal scute notches, which correspond to a unique identification number assigned in previous years from various projects within the lab [12]. Adult turtles who were not marked previously were notched with a Dremel^®^ 200 Two Speed Rotary Tool kit, while unmarked juveniles were notched with a standard square file. All new captures were given a new identification number. Turtles were sexed based on size and cloacal position. Turtles unable to be sexed due to size and placement of the cloaca were assigned “N/A”. Age class was determined by size and cloacal position, as juveniles are similar in size to adult male map turtles. Growth lines on carapacial scutes were also counted to estimate age. Microbiome samples were taken via separate cloacal and oral swabs using Puritan^®^ sterile rayon tipped swabs. Swabs were inserted into the cloaca or oral cavity and rotated for 30 s, with two swabs being taken simultaneously per sample type. Hatchlings did not receive a cloacal swab due to size. Field blanks were not collected due to our strict contamination reduction methods. Samples were then placed in a tube with 95% ethanol and placed in an −80 °C freezer until further processing. Individuals were then released to their original capture location.

### Sample Preparation & Sequencing

Swabs were removed from 95% ethanol and left to dry overnight in a sterile hood. DNA was then extracted using the ZymoBIOMICS™ DNA miniprep kit and then placed into a −80℃ freezer until sequencing. Samples were placed on dry ice and shipped to SeqCenter in Pittsburgh, Pennsylvania. The V3/V4 regions of the 16 S rRNA gene were targeted using the forward primers CCTACGGGDGGCWGCAG and CCTAYGGGGYGCWGCAG (341f) and the reverse primer GACTACHVGGGTATCTAATCC (806r) with the ZymoBIOMICS™ Quick-16 S™ NGS library preparation kit (Zymo Research, Irvine, CA) by SeqCenter. Library preparation occurred as described in the Zymo protocol book. Briefly, a 96-well PCR plate was loaded with our DNA samples into their respective wells, with one well containing 2µL of the ZymoBIOMICS™ Microbial Community DNA Standard as a positive control. One well contained only the master mix, which acted as the negative control. Index primer set A was used during the Barcode Addition step. During library quantification and pooling, all libraries and samples were normalized and 5µL of each were pooled to create the library loaded onto the sequencer. Samples were then sequenced on a P1 600cyc Illumina NextSeq2000 Flowcell to generate 2 × 301 bp paired-end reads.

### Data Processing

Sequences were demultiplexed by SeqCenter and received as paired-end reads, which were merged using QIIME2 (version 2023.2) [31]. Primer sequences were trimmed using the CutAdapt plugin within QIIME2 in order to improve read quality. Sequences were then denoised using the DADA2 plugin, where the reverse reads were truncated at the 256 position. The resulting feature table containing the amplicon sequence variants (ASVs) within all of our samples was then rarified. Alpha and beta diversity analyses were then conducted to confirm that cloacal and oral microbiomes should be separated in subsequent analysis. The original DADA2 table was then filtered by sample type into two separate tables so that cloacal and oral microbiome samples can be analyzed independently from one another. Both tables were separately rarefied and then used for alpha and beta diversity analyses.

### Taxonomic Classification & Differential Abundance

A phylogenetic tree was constructed by referencing our sequences to the Greengenes2 (version 2022.10) database in QIIME2 and taxonomic classifications were assigned. These taxonomic groupings were visualized with a bar chart in QIIME2, which also displayed the relative abundances of these taxa at the phylum and family level. The most abundant phyla and families across all samples were considered to be part of the core microbiome. Barplots were customized using the QIIME2R and ggplot2 packages in RStudio version 4.4.1 [32–34]. We also filtered taxa that were identified at a genus or species level and were present 80% of our samples in QIIME2, which we classified as core features within the microbiome. All prokaryotic names were validated using the List of Prokaryotic names with Standing in Nomenclature (LPSN) database [35]. Taxonomic results are presented at multiple levels in order to compare our results to other studies.

To determine what taxa were significantly depleted or enriched between groups, we conducted an analysis of compositions of microbiomes with bias correction (ANCOM-BC) in QIIME2 [36]. Comparisons were made between sample types, sides of the dam, and sex. Tests were run at the phylum, family, and feature level for all groups. Visualization occurred within QIIME2. Differentially abundant bacterial taxa were then searched on Google Scholar to assess potential function or source. Results were then compiled into a table.

### Diversity Analyses

α-diversity was measured using Shannon Index and Faith’s phylogenetic diversity (PD) [37] in QIIME2 at the feature level. Both metrics were statistically analyzed using a Kruskal-Wallis test with a Benjamini & Hochberg correction. We assessed differences among swab type, sides of the dam, and sex. Data were visualized as boxplots using the phyloseq, QIIME2R and ggplot2 packages in RStudio [31, 32, 34, 37].

β-diversity was measured using Bray-Curtis dissimilarity and Weighted Unifrac distance [37] in QIIME2 at the feature level. Microbiome community differences between sides of the dam were statistically examined using a PERMANOVA test with 999 permutations. Community differences between the two sides of the dam, swab type, and sex were examined. If the PERMANOVA yielded a significant p-value (*p* < 0.05), a PERMDISP analysis was conducted to determine if the significance was due to multivariate dispersion. Bray-Curtis and Weighted Unifrac results were visualized with a Principal Coordinates Analysis (PCoA) plot in RStudio using the phyloseq, QIIME2R and ggplot2 packages [32–34, 38]. Statistical comparisons between individual study sites were conducted for both α and β diversity and can be seen in the supplemental material.

## Results

### Field Results

A total of 35 cloacal and 33 oral swabs were collected over the sampling period. 18 cloacal swabs were collected above the Conowingo dam, while 17 cloacal swabs were collected below the dam. Of these, 20 cloacal samples were collected from females, 7 from males, and 8 from individuals of an undetermined sex. 18 oral swabs were collected above the dam, and 15 oral samples were collected below. Of these, 17 were collected from females, 6 from males, and 10 from individuals of an undetermined sex. Full field results can be seen in the supplementary material as Table S1.

### Sequencing Results

A total of 17,401,371 paired-end reads were present across all samples (*n* = 68) after demultiplexing. After denoising, 8,344,166 reads were present with a minimum frequency of 80,606. Samples were then rarified to 80,000 reads, where ~ 65% (5,440,000) of these reads were retained. 13,436 features were identified across all samples after rarefication. This encompasses 58 phyla, 129 classes, 360 orders, 652 families, 1404 genera, and 1385 species.

### Cloacal vs. Oral Microbiomes

*Pseudomonadota* (synonym: Proteobacteria), *Bacteroidota* (synonym: Bacteroidetes), and *Bacillota* (synonym: Firmicutes) were the top three most abundant phyla across both sample types (Fig. 1A). *Pseudomonadota* (synonym: Proteobacteria) was the most abundant phylum, with a 43.88% frequency for oral samples and 39.71% for cloacal. *Bacteroidota* (synonym: Bacteroidetes) had a 15.38% frequency for oral samples and 19.63% for cloacal samples. *Bacillota* (synonym: Firmicutes) had a frequency of 13.95% for oral samples and 10.43% for cloacal samples. Other abundant phyla include *Deinococcota* (synonym: Deinococcus-Thermus), *Actinomycetota* (synonym: Actinobacteria), *Cyanobacteriota* (synonym: Cyanobacteria), *Chloroflexota* (synonym: Chloroflexi), and *Acidobacteriota* (synonym: Acidobacteria) (Fig. S2). Of these, only *Pseudomonadota* (synonym: Proteobacteria) (*p* = 0.003) and *Deinococcota* (synonym: Deinococcus-Thermus) (*p* = 0.02) (see ANCOM results supplemental file) showed a significant difference in abundance between sample types, with these two phyla being particularly enriched in oral samples compared to cloacal samples. *Enterobacteriaceae*,* Bacillaceae*, and *Weeksellaceae* were the top three most abundant at the family level for both sample types. Other common families include *Burkholderiaceae*,* Deinococcaceae*,* Paracoccaceae* (synonym: Rhodobacteraceae), *Flavobacteriaceae*,* Sphingomonadaceae*, and *Lysobacterceae* (synonym: Xanthomonadaceae) (Fig. 1B). *Lysobacterceae* (synonym: Xanthomonadaceae), *Listeriaceae*,* Burkholderiaceae*,* Hyphomonadaceae* and *Lentimicrobiaceae were* all significantly depleted in cloacal samples compared to oral. Full results for the differential abundance analysis between sample types can be seen in the supplemental material. Features with an 80% prevalence across our samples are summarized in Table 1, which were considered as the core features.

**Fig. 2:**
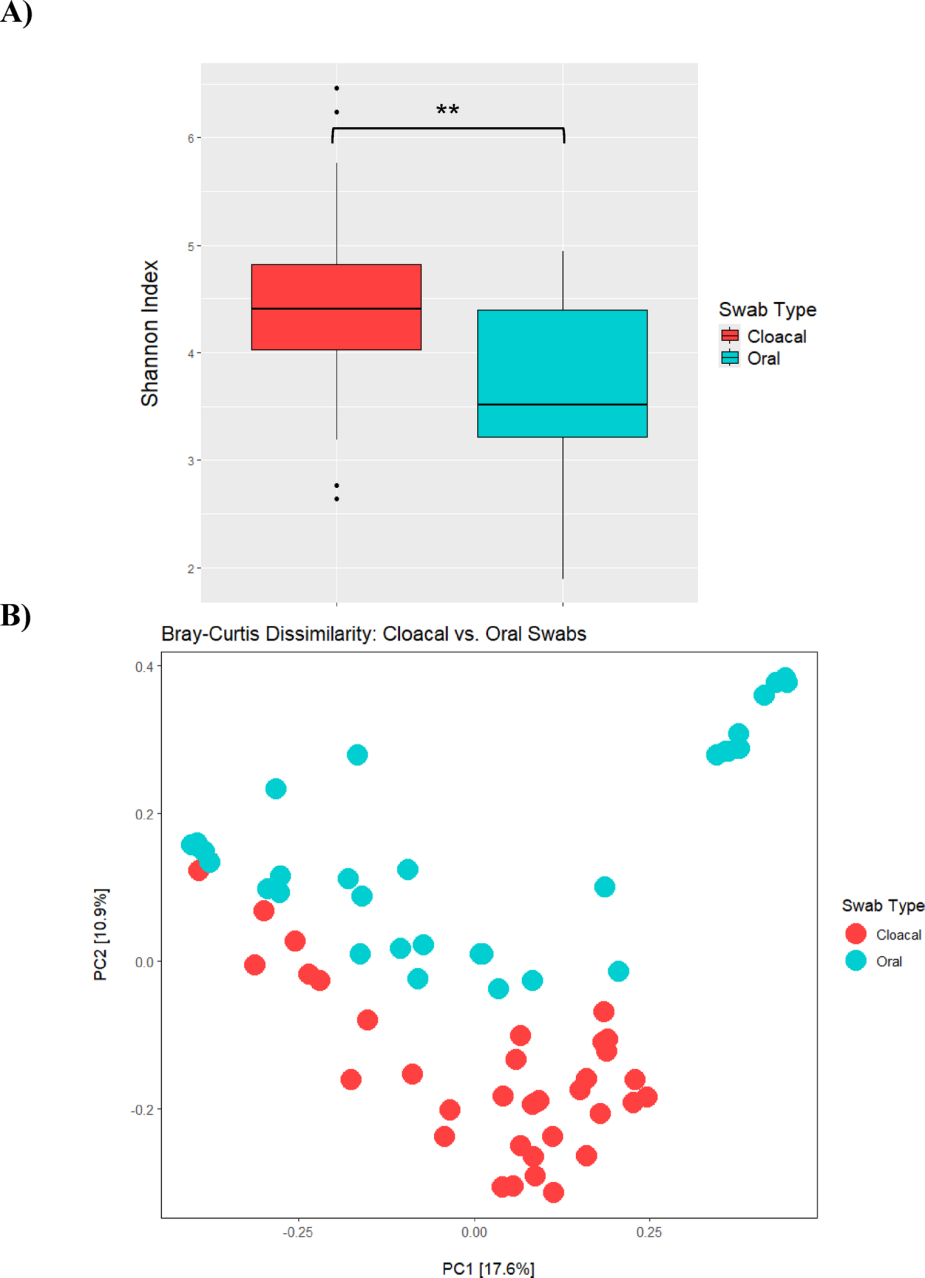
Diversity metrics comparing cloacal (*n* = 35) and oral (*n* = 33) samples. **A** Shows the Shannon Index, where diversity was significantly different between the two sample types (*p* = 0.004). **B** Shows Bray-Curtis dissimilarity, which was statistically significant (*p* = 0.001) between the two sample types

Alpha diversity analyses showed that bacterial diversity was significantly different between cloacal and oral microbiomes for the Shannon Index (Kruskal-Wallis; *H* = 12.44, *p* = 0.0004) and Faith’s PD (Kruskal-Wallis; *H* = 5.18, *p* = 0.022) (Fig. S2). Cloacal microbiomes had a higher diversity (Fig. 2B) for both metrics. Full alpha diversity results can be seen in in the Supplemental Files.

Beta diversity analyses showed that microbiome community composition was distinct between cloacal and oral samples when measuring both Bray-Curtis (PERMANOVA; *F* = 5.42, *p* = 0.001) (Fig. 2B) and Weighted Unifrac (PERMANOVA; F = 4.13, *p* = 0.002) (Fig. S2). These differences were not found to be due to multivariate dispersion for Bray-Curtis (PERMDISP; *F* = 1.16, *p* = 0.272) or Weighted Unifrac (PERMDISP; *F* = 0.93, *p* = 0.326). Full beta diversity results can be seen in Table S3. The statistical differences between cloacal and oral microbiomes confirmed that sample types should be separated in subsequent analyses.

**Table 1:** Summary of core features for all samples. Core features were defined as taxa that could be identified at the genus or species level and were present in 80% of all samples

Swab type	Classification	Name	Notes	Source
Cloacal	Genus	*Bdellvibrio spp.*	Common in the environment, and isolated from mammalian feces. Has antibiotic properties; consumes other harmful bacteria.	[39]
		*Dethiosulfovibrio spp.*	Reduces sulfur and thiosulfate. Can be found in the environment and as a part of healthy human gut microbiota. May be opportunistic pathogens. Can corrode iron and steel, contribute to mercury methylation in soil, and remediation of petroleum byproducts.	[40]
		*Novosphingobium spp.*	Indicator of metal contamination.	[41]
		*Streptococcus spp.*	Common pathogenic group, associated with septicemia; has been isolated from lesions in Sea turtles, especially in the digestive tract.	[42, 43]
		*Inhella spp.*	Has been found on the eggshells of Amazon River turtles, increasing over the course of egg development.	[44]
		*Corynebacterium spp.*	Has been found in soil and water, including species capable of degrading sludge and squalene. Associated with various diseases in reptiles, including chelonians. Can be part of regular microbiota in birds. May be a source of zoonotic disease.	[42, 45]
		*Aquabacterium spp.*	Originally isolated from drinking water. Has the ability to degrade plastic polymers.	[46)
		*Herpetosiphon spp.*	Has been isolated from freshwater and soil sources. Possesses antimicrobial properties.	[47]
		*Pseudomonas spp.*	Commonly found in soil, water, and plants. May be associated with hatching success in sea turtles. Some species have been linked to dermatitis in turtles.	[42, 48]
		*Sphingomicrobium spp.*	Isolated from sea water. Associated with marine environments.	[49]
		*Flavobacterium spp.*	Found in soil and freshwater environments. Several species cause diseases in fish. Has been found in the buccal cavity of Krefft’s river turtles (*Emydura macquarii krefftii*). One species has been isolated from the liver of a deceased Barbour’s map turtle (*Graptemys barbouri*).	[50–52]
		*Ferruginibacter spp.*	Isolated from freshwater sediments. Found to be significant for wastewater treatment plants.	[53, 54]
Both	Genus	*Deinococcus spp.*	Resistant to environmental stress, radiation, and other extreme conditions. Can inhibit growth of *Staphylococcus aureus.* Found in the cloaca of shorebirds.	[55, 56]
		*Sphingomonas spp.*	Found in both contaminated and uncontaminated environments. Can help plant growth and stress tolerance. Plays a role in degradation of various xenobiotic compounds such as polycyclic aromatic hydrocarbon (PAH), estradiol, herbicides, pesticides, biphenyls, and heavy metals.	[57]
		*Massilia spp.*	Have been isolated from various soil habitats and human blood. Some can produce the antibiotic violacein. May play a role in phenanthrene (a PAH) degradation.	[58, 59]
		*Paracoccus spp.*	Found on the carapace of several freshwater turtle species across various habitats. Considered to be a metabolically flexible genus. Some species are capable of xenobiotic degradation and have been isolated from polluted environments.	[60, 61]
		*Acinetobacter spp.*	Can indicate pollution/poor water quality and associated with detoxifying heavy metals. Could be associated with stomatitis or pharyngitis in turtles. Previously studied in Amazon turtle (*Podocnemis expansa*)	[41, 42, 62]
		*Bacillus spp.*	Commonly present in soil/water. Many can be pathogenic in sea turtles and softshell turtles. *Bacillus cereus*, a source of food poisoning, has been isolated from Wood Turtle feces.	[42, 43, 63, 64]
		*Staphylococcus spp.*	Commonly present on the mucosal membranes of animals. Many are opportunistic pathogens. Has been studied in turtles as a source of disease.	[43, 65]

### The Impact of the Dam on Map Turtle Microbiota

Cloacal microbiome diversity was significantly different based on relativity to the dam (above vs. below) using the Shannon index (Kruskal-Wallis; H = 5.19, *p* = 0.023), with sites below the dam having a higher diversity (Fig. 4A). Oral microbiome diversity did not vary significantly based on location relative to the dam when using the Shannon index (Kruskal-Wallis; H = 0.52, *p* = 0.47). The dam was significant in determining Faith’s PD for oral microbiomes (Kruskal-Wallis; *H* = 8.16, *p* = 0.004), but not cloacal (Kruskal-Wallis; *H* = 1.41, *p* = 0.235) (Fig. 4B).

Cloacal microbiome community composition varied significantly between the two sides of the dam for both Bray-Curtis (PERMANOVA; *F* = 1.99, *p* = 0.006) (Fig. 5A) and Weighted Unifrac (PERMANOVA; *F* = 2.32, *p* = 0.014) (Fig. S3). However, the significance of the Weighted Unifrac results could be due to multivariate dispersion (Table S3). Oral microbiome community composition also varied significantly based on location relative to the dam for both Bray-Curtis (PERMANOVA; *F* = 6.87, *p* = 0.001) (Fig. 5B) and Weighted Unifrac (PERMANOVA; *F* = 9.06, *p* = 0.002) (Fig. S3). The significance of the Bray-Curtis dissimilarity results could also be due to multivariate dispersion (Table S3).

**Fig. 3:**
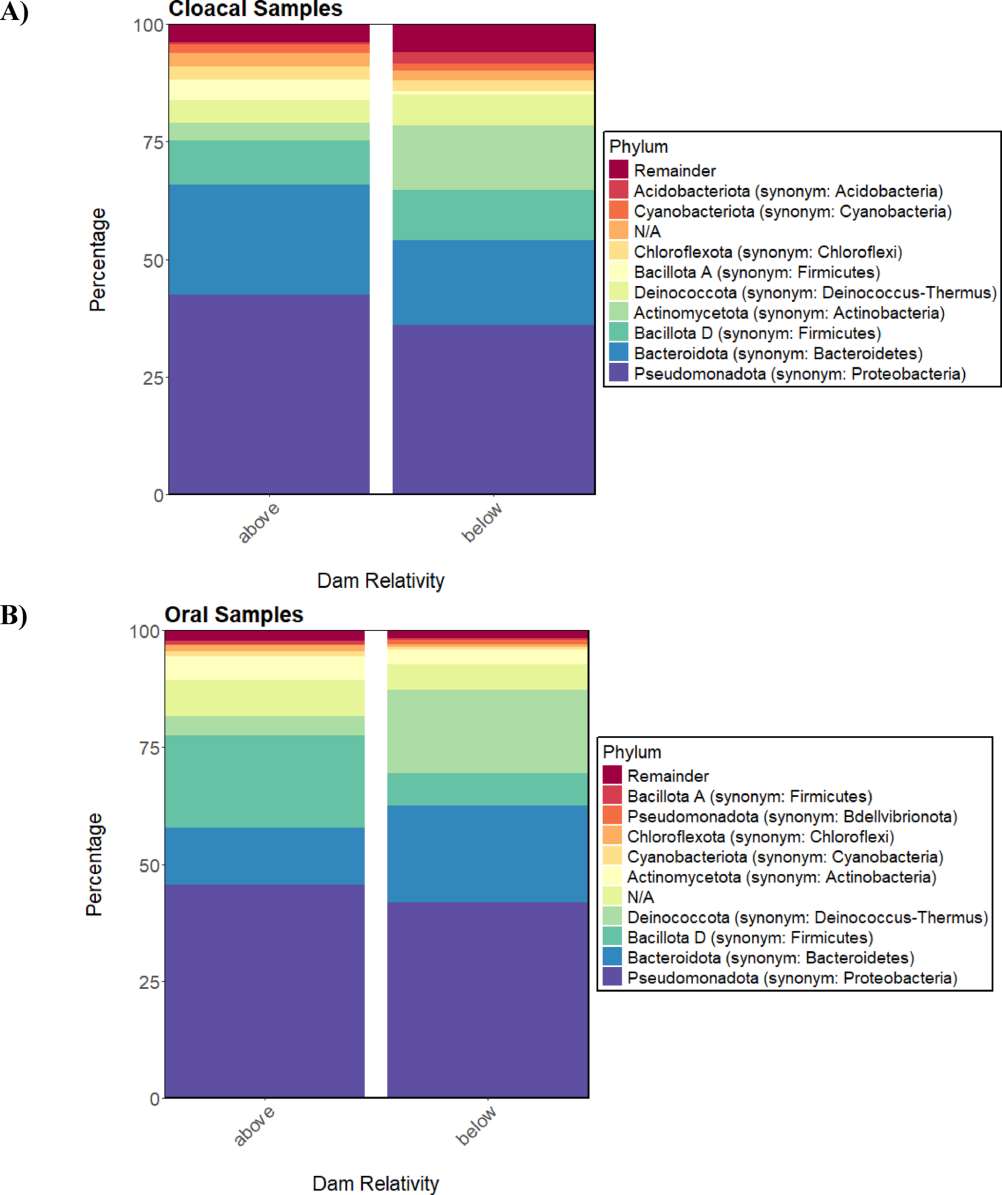
Percentage of bacterial phyla in both sample types by location relative to the hydroelectric dam. **A** Shows cloacal samples, with above (*n* = 18) on the left and below (*n* = 17) on the right. **B** Shows oral samples, with above (*n* = 18) on the left and below (*n* = 15) on the right. “N/A” are the proportion of taxa that have not yet been identified at that level. “Remainder” indicates the proportion of taxa that have been identified but are in lower abundance

ANCOM-BC differential abundance analysis of cloacal samples revealed that the phyla Pseudomonadota (synonym: Proteobacteria), Bacteroidota (synonym: Bacteroidetes) and Fusobacteriota (synonym: Fusobacteria) were significantly enriched in turtles above the dam compared to those below (Fig. 3). In oral samples, Bacillota (synonym: Firmicutes) was the only phylum that was significantly enriched in turtles above the dam compared to below, while Bacteroidota (synonym: Bacteroidetes) and Deinococcota (synonym: Deinococcus-Thermus), were significantly enriched in those below the dam (Fig. 3). Features that were significantly enriched above the dam compared to below in our ANCOM-BC analysis can be observed in Table 2. Full ANCOM-BC results can be found among the supplemental files.

**Table 2:** Bacterial taxa that were significantly enriched in samples above the dam compared to below, which may be associated with the conditions created by the dam. Results are based on ANCOM-BC analysis

Swab type	Classification	Name	Notes	Source
Cloacal	Phylum	Thermoproteota	Typically found in acidic waters with high temperatures.	[66]
		Desulfobacterota	Particularly abundant in black and odorous water, which is a product of pollution. May be toxic. Has been identified in Chinese striped-neck turtle (*Mauremys sinensis*), which decreased after the exposure of ammonia.	[67, 68]
	Family	*Tannerellaceae*	Indicator of fecal contamination in urban lakes.	[69]
		*Comamonadaceae*	Associated with sewage sludge.	[70]
		*Aeromonadaceae*	Some cause disease in turtles; may also be associated with sewage sludge but can be found regularly in freshwater	[42, 71]
		*Sphingomonadaceae*	Significant in Triclosan degradation, which is a common commercial antibacterial/antifungal agent.	[72]
		*Saprospiraceae*	Common in wastewater treatment plants.	[73]
		*Microscillaceae*	Associated with Microcystis, a genus of cyanobacteria responsible for algal blooms.	[74]
		*Bacillaceae*	Commonly found in contaminated and polluted environments, especially those with hydrocarbons and heavy metals. Can inhibit sulfate-reducing bacteria.	[75]
		*Desulfovibrionaceae*	Reduces sulfate in low oxygen environments.	[76]
	Genus	*Cloacibacterium spp.*	Has been isolated from naphthalene contaminated water. Associated with polycyclic aromatic hydrocarbons (PAHs)	[77]
		*Aeromonas spp.*	Opportunistic pathogen in chelonians, linked to dermatitis and stomatitis	[43]
		*Accumulibacter spp.*	Known for its ability to reduce phosphorus, particularly in wastewater systems.	[78]
		*Clostridium spp.*	Associated with various diseases in turtles, including shell disease in map turtles	[41, 43, 80, 117]
		*Kaistella spp.*	Has been isolated from freshwater streams and soils, with one isolate from oil-contaminated soil.	[79]
		*Proteiniphilum spp.*	May be associated with oil contamination.	[81]
		*Streptomyces spp.*	May play a role in petroleum degradation	[82]
	Species	*Acinetobacter indicus*	Isolated from hexachlorocyclohexane (insecticide) dump site. Has also been isolated from an urban sewage treatment plant.	[83, 84]
		*Brevundimonas diminuta*	May indicate arsenic contamination.	[85]
		*Cetobacterium somerae*	Commonly found in the microbiome of fish in freshwater ecosystems. Could also be associated with plastics or Perflurooctanic acid exposure.	[86, 87]
Oral	Phylum	Nitrospira	Contributes to nitrification of aquatic sediments. Can also metabolize sulfur	[89]
		Planctomycetota	May be significant source of antibiotic resistance genes in the environment.	[90]
		Gemmatimonadota	Commonly found in activated sludge from wastewater treatment plants. Some are capable of anoxygenic photosynthesis.	[91]
		Bdellovibrionota	Known for its ability to degrade pollutants, particularly phthalic acid esters (PAEs)	[92]
	Family	*Enterobacteriaceae*	Several species can cause disease in reptiles	[42]
		*Burkholderiaceae*	Opportunistic pathogen that is resistant to disinfectants and was found to be enriched in water bodies surrounding areas with heavy livestock.	[93]
	Genus	*Neisseria spp.*	Can cause infections, including oral abscess formation in iguanas	[42]
		*Bacillus spp.*	Commonly present in soil/water. Can be associated with heavy metal contamination. can be pathogenic in sea turtles and softshell turtles	[41–43, 62]
		*Acinetobacter spp.*	Can indicate pollution/poor water quality and associated with detoxifying heavy metals.	[41]
Both	Genus	*Aquamicrobium spp.*	Several species have been isolated from contaminated soils.	[94, 95]
		*Parabacteroides spp.*	May indicate minor Perfluorooctanic acid (PFOA) exposure.	[88]

**Fig. 4:**
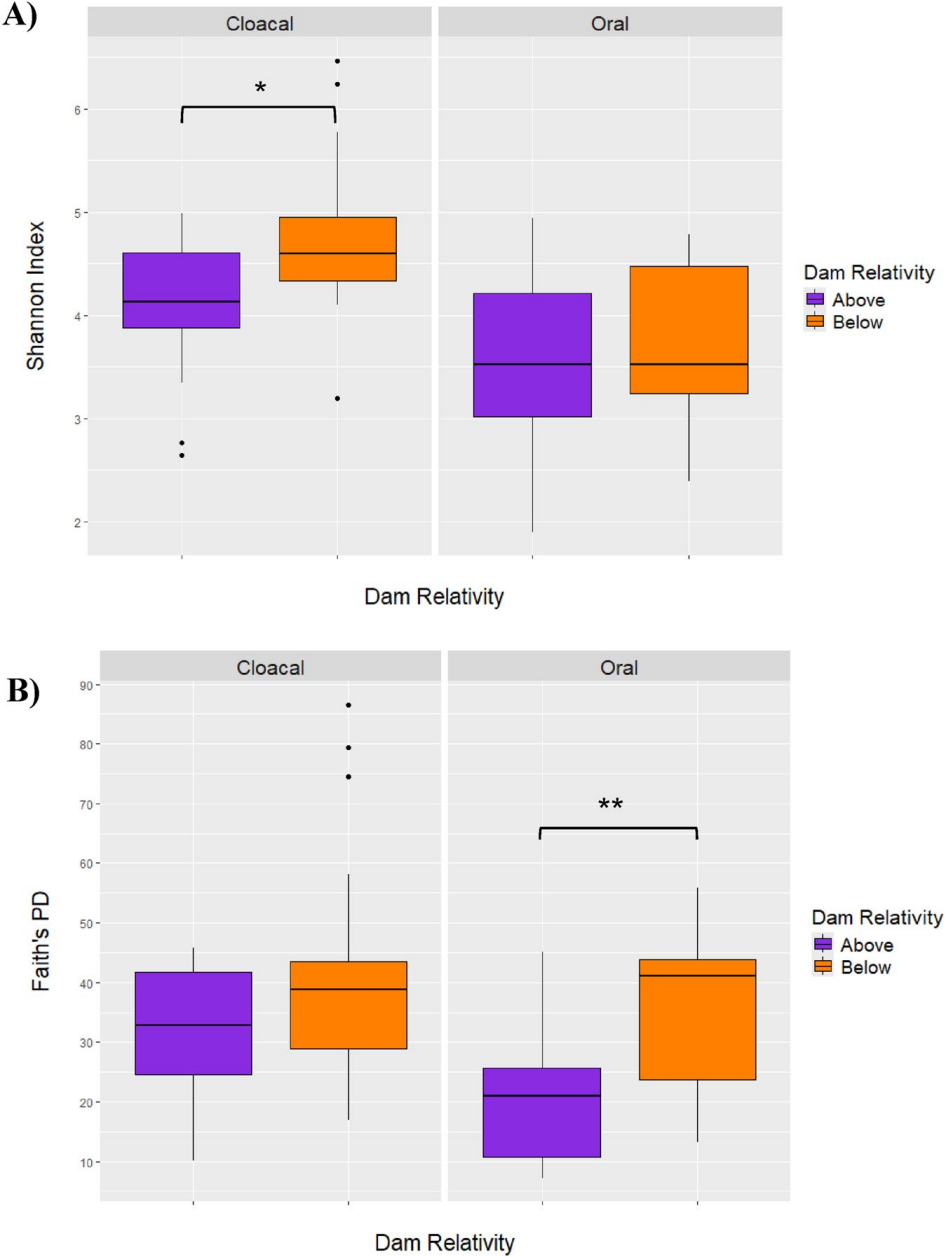
Alpha diversity metrics for cloacal (*n* = 35) (left) and oral (*n* = 33) (right) swabs compared by relativity to the dam. **A** Shows the Shannon Index, which was significantly different for cloacal (*p* = 0.02), but not oral (*p* = 0.47) samples. **B** Shows Faith’s Phylogenetic Diversity, which was significantly different for oral (*p* = 0.004), but not cloacal (*p* = 0.23) samples

**Fig. 5:**
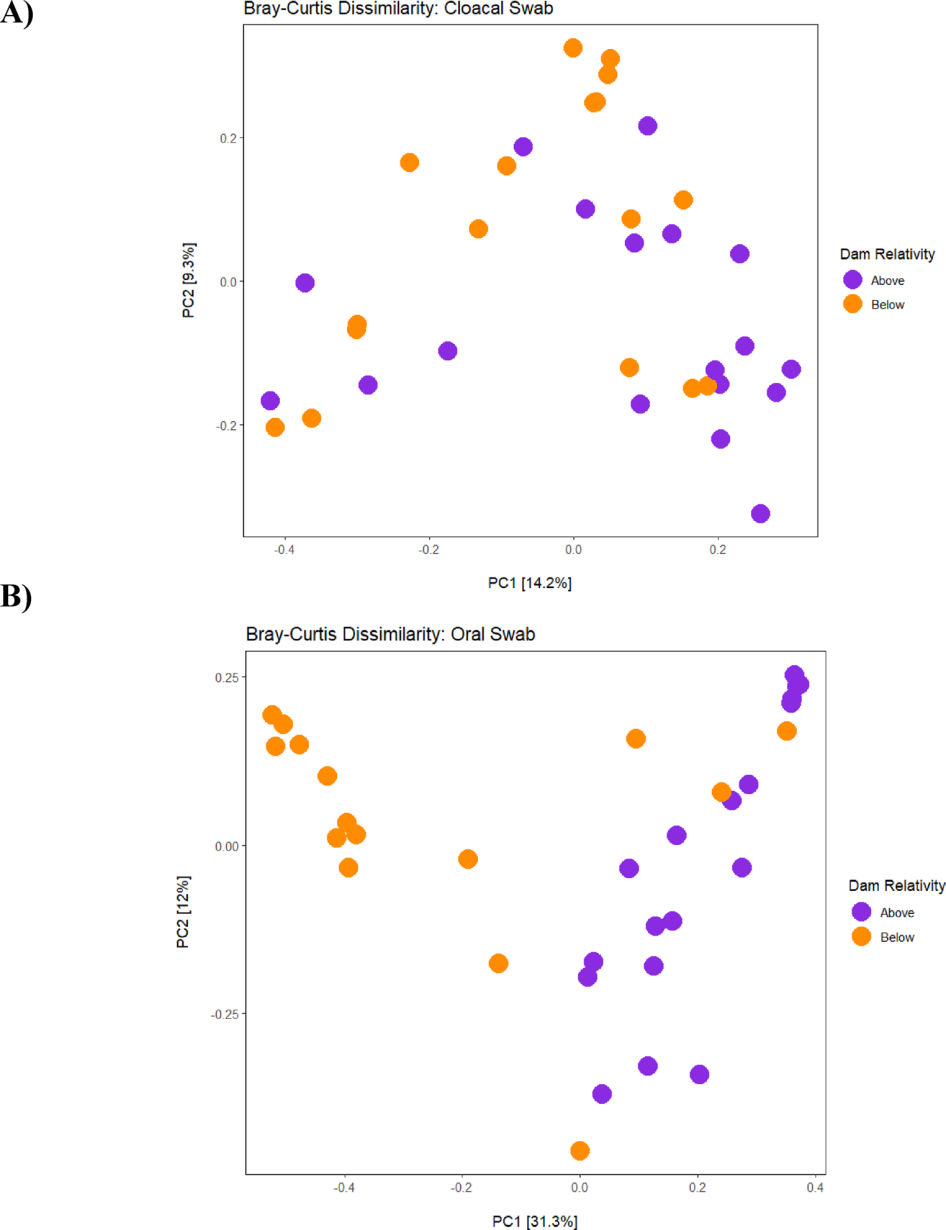
Bray-Curtis dissimilarity ordination plots for both sample types based on location relative to the dam, with samples above the dam denoted by purple and those below denoted by orange. **A** Shows cloacal samples, where there was statistical significance between the two locations (*p* = 0.006). **B** Shows oral samples, where there was also a statistical significance (*p* = 0.001) between the two locations

### Sex Differences in Microbiota

Cloacal and oral microbiome alpha diversity analyses yielded insignificant results when comparing males and females for all metrics (Table S2). Beta diversity was significant between males and females for all metrics across all samples (Table S3). ANCOM-BC analysis revealed no notable dietary-associated taxa that were significantly different between the two sexes for either sample type. See supplementary files for full results.

## Discussion

In this study, we characterized the cloacal and oral microbiomes of a population of Northern Map Turtles that is fragmented by a large hydroelectric dam in a major river impacted by urbanization. We focus on how the presence of the dam affects microbiome structure, addressing differences in specific bacterial taxa between the two sides. We compared the composition of cloacal and oral microbiomes and established a core microbiome for this population, which we classified as bacteria that were heavily abundant across both sides of the dam. Lastly, we evaluated potential differences between male and female Northern Map Turtle microbiomes.

### Cloacal vs. Oral Microbiomes

Our results showed that cloacal and oral microbiomes in this population of Northern Map Turtles (*Graptemys geographica*) differ in composition and diversity. Cloacal swabs are a minimally invasive and relatively quick way to study the gut microbiota in turtles [96, 97], as fecal samples can be difficult to obtain in the wild [98] and collecting gut samples would require euthanasia and necropsy. Oral swabs may represent environmentally associated microbes in turtles [50] and other freshwater reptiles [91]. Oral microbiomes are not well understood in aquatic freshwater turtles, making the present study one of the few to study it in these species. This is also the first study that compares cloacal and oral microbiomes in a freshwater turtle species, as previous studies in freshwater turtles have only focused on sampling one body area [60, 96] or comparing external surfaces to the oral cavity [50]. We found that cloacal microbiomes are significantly higher in alpha diversity compared to oral microbiomes (Fig. 2A), which is notable considering that the swabs were taken from the same individuals. Our results contradict a previous study in Kemp’s ridley sea turtles (*Lepidochelys kempii*), that found that oral microbiomes were higher in alpha diversity [99]. Another study in loggerhead sea turtles (*Caretta caretta*) found no difference in cloacal and oral microbial diversity, likely due to a relatively small target population [100]. The disparity between marine and freshwater turtle microbiomes is likely related to differences in habitat and diet, as a study in the semi-aquatic water monitor lizard (*Varanus salvator*) also found that their cloacal microbiomes were significantly higher in alpha diversity compared to oral [101]. Both habitat and diet are known to be drivers of alpha diversity differences across reptile taxa [102], so it is unsurprising that there are discrepancies between marine and freshwater turtle species.

Separation of cloacal and oral samples can be observed in Fig. 2B, with some overlap in Weighted Unifrac (Fig. S2). The slight similarity of cloacal and oral samples for this metric may be explained by the fact that the two surfaces of the two sample regions are both in constant contact with the environment and thus may share some specific features [96]. However, differences in cloacal and oral microbiome composition are further emphasized by our differential abundance analyses. Here, we found several taxonomic groups that were significantly enriched or depleted in cloacal samples compared to oral (See Supplemental File). Several of these taxa are associated with the environment and were enriched in oral samples compared to cloacal, such as *Undibacterium*, *Flavobacterium cheniae*,* Fluviicola*, and *Agitococcus lubricus* [103–106]. The high prevalence of these features in the oral cavity shows a close association of oral microbiota to the environment, consistent with what is expected. To our knowledge, this is the first study to examine the microbiota of Northern Map Turtles. These results suggest that future studies in this species should separate cloacal and oral microbiomes in their analyses.

### Core Microbiome

We were able to identify the most abundant bacterial taxa within the microbiomes of this population of Northern Map Turtles, which, to our knowledge, has never been studied in this species. *Pseudomonadota* (synonym: Proteobacteria), *Bacillota* (synonym: Firmicutes), and *Bacteroidota* (synonym: Bacteroidetes) were the most abundant phyla for both sample types, which have been previously identified as the core phyla across all reptiles [107]. The dominance of *Pseudomonadota* (synonym: Proteobacteria) in our samples is consistent with a previous study in the closely related False Map Turtle (*Graptemys pseudogeographica*), which found that *Pseudomonadota* (synonym: Proteobacteria) was the most abundant in the cloacal microbiome across various sites [97]. The oral microbiome had a significantly higher abundance of *Pseudomonadota* (synonym: Proteobacteria) in our study, which has previously been shown to be particularly abundant in the buccal cavity of Krefft’s river turtle (*Emydura macquarii krefftii*) [50]. The study in False Map Turtles found that the phyla *Deinococcota* (synonym: Deinococcus-Thermus), *Actinomycetota* (synonym: Actinobacteria), and *Chloroflexota* (synonym: Chloroflexi) were abundant in their samples [89], which we also found in our results (Fig. 1A). Only *Deinococcota* (synonym: Deinococcus-Thermus) was significantly different between our sample types, being more abundant in the oral cavity. This phylum is known for surviving in extreme environments and is highly resistant to stress [108]. It was found to be highly abundant in the gut of wild crocodile lizards (*Shinisaurus crocodilurus*) compared to captive, suggesting that this phylum may help with environmental adaptation in freshwater reptiles [109]. A previous review suggested that host phylogeny is a significant determinant of microbiome diversity in reptiles [102], so comparisons between reptilian orders should be taken lightly.

*Acidobacteriota* (synonym: Acidobacteria), and *Cyanobacteriota* (synonym: Cyanobacteria) were also abundant in our samples (Fig. 1). *Cyanobacteriota* (synonym: Cyanobacteria) is ubiquitous in the environment and was found to be abundant on external body areas of freshwater turtles [50]. *Acidobacteriota* (synonym: Acidobacteria) is also associated with the environment, being particularly enriched in soil and aquatic sediments [110]. The high abundance of both *Cyanobacteriota* (synonym: Cyanobacteria) and *Acidobacteriota* (synonym: Acidobacteria), in our samples further suggests that Northern Map Turtles have some level of microbial acquisition from their habitat. The core families in our study included *Enterobacteriaceae*,* Bacillaceae*, and *Weeksellaceae* (Fig. S2B). *Weeksellaceae* has been found to be abundant in both the oral cavity of Krefft’s river turtles (*Emydura macquarii krefftii*) [50] and the cloaca of False Map Turtles (*Graptemys pseudogeographica*) [89]. *Enterobacteriaceae* encompasses several species that are associated with disease, both in chelonians and as potential sources for zoonotic transmission to humans [42, 43].

We were also able to identify core features, which we defined as taxa that could be identified at the genus or species level and had an 80% prevalence across all samples for both cloacal and oral swabs (Table 1). Some of these features may be beneficial for immunity and resistance to stress, such as *Bdellvibrio. Herpetosiphon*,* Massilia* and *Deinococcus* [39, 47, 55, 58, 59]. However, several features found in both sample types were associated with pollution degradation and poor water quality, including *Acinetobacter* and *Sphingomonas* [41, 57]. *Novosphingobium* was highly prevalent in cloacal samples, which is also associated with pollution [41]. Both *Acinetobacter* and *Novosphingobium* are associated with metal contamination and were increased in water following the collapse of a dam in Brazil [41]. Metal contamination has been measured in the Lower Susquehanna, with mercury, selenium and lead being trapped in sediments from mostly anthropogenic sources [111]. Despite Conowingo Dam historically having the lowest concentration of mercury in sediment compared to other dams in the Susquehanna [111], the presence of *Sphingomonas* may suggest increased levels of mercury since that study, as it is specifically associated with its degradation [57].

*Sphingomonas* [57] and *Massilia* [59] may also play roles in polycyclic aromatic hydrocarbon (PAH) degradation in this system (Table 1). PAHs are associated with the burning of petroleum and coal and have been found in high concentrations within the Conowingo Reservoir [112]. Potential sources of these PAHs include urban storm runoff, mining operations, ash from power plants, and automobile emissions [113]. The frequent presence of these microbes may suggest a widespread amount of PAHs exposure, as they are known to grow in the presence of hydrocarbons [53, 55]. This is of concern because PAHs have been shown to negatively affect development, hatching success, and survival in other freshwater turtle species [113]. The prevalence of taxa associated with pollution emphasizes a further need for water quality and other management practices to be implemented in this system.

### The Conowingo Dam & Map Turtle Microbiota

The dam was an important factor in determining microbiome diversity and composition for both sample types. Cloacal microbiomes were significantly more diverse in samples below the dam when measuring the Shannon Index, but not Faith’s PD (Fig. 4). This could mean that there are differences in the abundance of taxa between turtles on opposite sides of the dam, but that these taxa are not necessarily phylogenetically distinct from one another [114]. However, both boxplots showed that cloacal microbiomes in turtles below the dam had a higher median alpha diversity for both metrics (Fig. 4). A previous study comparing the gut microbiome of various fish species upstream and downstream of a hydroelectric dam found that fish at sites that upstream of the dam had a higher alpha diversity [15], which is opposite of our results. To our knowledge, this is the only other study that compares microbiota of wildlife living in a dam-impacted system. The difference in cloacal microbiome diversity upstream and downstream of the dam may be explained by the differences in prey base [24] but may also indicate differences in anthropogenic pressure between the two sides [115]. The significant differences in cloacal microbiome diversity because of the dam raises conservation concern, as decreases in diversity are often linked to dysbiosis and can have long-term implications on host health [116]. These differences can also be reflective of overall ecosystem health and represent an ecological disturbance [6]. Both measures of beta diversity showed that cloacal microbiome composition was also significantly different between sides of the dam (Fig. 5A). Given the lack of knowledge of how dams affect gut microbiota, more work needs to be done to understand these differences and how they will affect long-term health of Northern Map Turtles and other wildlife.

Oral microbiomes had a significantly higher Faith’s PD below the dam compared to above, while Shannon Index was not significantly different (Fig. 4). This suggests that oral microbiomes below the dam have a significantly higher amount of phylogenetically distinct taxa, but that there is not a huge difference in their abundance [114]. Beta diversity analyses revealed that oral microbiomes were significantly different in composition between sides of the dam, but this may have been due to dispersion for Bray-Curtis (Table S3). Nonetheless, a clear separation of samples based on the dam could be seen on both PCoA plots (Fig. 5B). This is consistent with the idea that each side of the dam has environmental differences, including sediment composition, water quality, and other biotic factors.

Differential abundance analysis between sides of the dam for both sample types revealed several taxa of interest. Desulfovibrionaceae, a family known for its ability to metabolize sulfur at low levels of oxygen [76], was significantly more enriched in the cloacal microbiomes of turtles above the dam. Sediments accumulated behind the dam contain low amounts of oxygen and sulfur [108], emphasizing the importance of this family in the environment. Similarly, Nitrospira was significantly enriched in oral samples above the dam. This phylum helps to nitrify aquatic sediments and can also metabolize sulfur. The existence of these taxa suggests that turtles above the dam may be ingesting sediments.

Some taxa that were significantly enriched in cloacal samples above the dam may be associated with water contamination, including Desulfobacterota, *Sphingomonadaceae*, *Comamonadaceae*,* Cloacibacterium*, *Brevundimonas diminuta*,* Cetobacterium somerae*,* Proteiniphilum*,* Streptomyces* and *Acinetobacter indicus* [67, 70, 72, 77, 81–85, 87] (Table 2). *Comamonadaceae* is associated with sewage sludge and has been identified in freshwater turtles before [115], which may suggest a higher prevalence of sewage runoff above the dam. *Acinetobacter indicus* has also been associated with sewage but is most known for being isolated from an insecticide dump site. *Brevundimonas dimunta* has been linked to arsenic contamination [85], and its high prevalence in cloacal samples above the dam is consistent with heavy metal trapping in dam sediment. *Cetobacterium somerae* has been linked to plastic pollution, as well as Perfluorooctanoic acid (PFOA), which is part of a class of emerging pollutants [87]. Interestingly, *Cloacibacterium* is specifically associated with PAHs, suggesting that there may be a higher concentration of PAHs in sediment above the dam [77]. The existence of these taxa within the cloaca of the turtles raises concern that these pollutants may be persistent in the environment and that the turtles are ingesting some amount of sediment and thus are accumulating them.

Various taxa associated with disease were also heavily enriched in cloacal samples of turtles above the dam, including *Aeromonadaceae*, *Aeromonas* and *Clostridium* [42, 43, 117] Species within *Aeromonas* and *Clostridium* have been associated with shell diseases in map turtle species [117] *Aeromonas spp*. are particularly known to be opportunistic pathogens across chelonians, causing dermatitis and stomatitis [42, 43]. The presence of these taxa could lead to infection if a turtle is injured or stressed, which could be a possibility with an urbanized population such as this. The potential relationship these taxa may have to healthy gut microbiota and disease in Northern Map Turtles in Maryland should be further explored.

Similarly, some taxa associated with pollution were also found to be differentially abundant in oral cavities above the dam, including Bdellvibrionota, *Bacillus spp.*, and *Acinetobacter spp.* (Table 2) [41–43, 62, 92]. Bdellvibrionota is known for its ability to degrade many pollutants, including phthalic acid esters (PAEs), which are released into the environment from plastic pollution [92]. The high abundance of taxa associated with pollution in turtles above the dam suggests that future conservation efforts for this population should focus on those sites. It also suggests that the presence of pollutants may have a negative impact on the turtles and their microbiomes.

We did not include analysis of individual study sites due to disproportionate sampling from logistical constraints in the field. Though we did see some statistical significance between study sites in our α and β analyses (see supplemental file), the uneven sampling may have led to false positives. We chose to focus on the dam because of the knowledge that the turtles can move between sites on the same side of the dam. Therefore, we cannot say that there is a difference in the microbiomes of turtles at each site.

### Sex Related Differences in Microbiota

Northern Map Turtles are known to have sexually dimorphic diets [24, 25], which may be reflected in our results. We found that the composition of microbiomes between the sexes was significantly different for both sample types, given our results from our beta diversity analyses (Table S3). Our plots showed some clustering of males and females (Figs. S4 & S5), and differentially abundant taxa could be identified for both sample types (See supplemental files). Prior studies in this population have shown little to no overlap in diet [24, 25], which is likely the reason for this separation. However, these differences could be due to the sampling bias of males above the dam and females below (Table S1). Though we were able to detect some differences between males and females in our beta diversity and differential abundance analyses, results from our alpha diversity revealed insignificant results between the two (Table S2). The lack of clear differences could also be explained by the fact males and females reside in the same environment. Additionally, we had disproportionately more females than males in our study, likely due to our study taking place during the nesting season. This likely affects the strength of these results, and more work needs to be done to understand the differences of microbiota between male and female Northern Map Turtles.

### Limitations

One limitation of comparing cloacal and oral microbiomes is that they are both mucosal surfaces that have frequent contact in the environment, so the cloacal microbiome is likely to somewhat represent the environment instead of the gut alone [96]. It is also important to note that the cloaca is an endpoint for the urinary and reproductive tracts, in addition to the digestive system [118]. In turtles, it may also play a role in respiration [118]. Therefore, microbes identified in this study may be associated with those systems. We also did not collect field blanks, and some of the microbes found may be directly from the environment instead of being host-associated. Though we were able to detect differences between cloacal and oral microbiota in Northern Map Turtles, more studies need to be done to understand the variation between them in other freshwater turtle species.

Our study was only focused on the bacterial microbiome by using 16 S sequencing and these results do not represent the whole microbiome. Future work should include other marker gene analyses such as ITS as well as shotgun metagenomics [37]. These results will provide a full scope of the fungi, small eukaryotes, and viruses that encompass the Northern Map Turtle microbiome.

It is also important to consider that our study examined just the V3/V4 region of the 16 s gene, which is not as accurate in identifying taxa to the species level compared to sequencing the entire gene [119] or supplementing with other gene analyses [120]. Therefore, all references to bacteria at the species level in this study should be considered preliminary until further analysis can be achieved. However, the V3/V4 region has been shown to be sufficient in identifying to the genus level [119] and can be considered correct to that extent in the present study. Additionally, functional predictions using 16 s rRNA sequencing can be poor performing and inconsistent among methods [121]. Thus, some of the inferences about the role of these taxa may not be exact and further research should be done to understand their functional role.

## Conclusion

Our results showed that the cloacal and oral microbiomes of Maryland’s Northern Map Turtles are significantly different in diversity and composition. We identified a core microbiome for this population, which appeared to be consistent with other reptile species. We also found some evidence to suggest that there are sex-related microbiome differences, which could be due to uneven sampling. To our knowledge, this study is the first to examine microbiomes of Northern Map Turtles. Therefore, these findings may provide insight for future studies involving this species.

Maryland’s population of Northern Map Turtles are classified as state-endangered and have been heavily impacted by urbanization. One potential threat they face is the Conowingo Dam, which played a large role in determining microbiome structure in our results. We found that cloacal and oral microbiomes are significantly different in composition and diversity between sides of the dam. Microbial communities were more diverse below the dam, and there were many bacterial taxa that were significantly different in abundance between the two sides. Several of these were associated with pollution or disease and were more abundant above the dam compared to below. Therefore, conservation focus should be on sites above the dam, which could include water quality monitoring, dredging, and habitat regulations. This dam is also one of several on the Lower Susquehanna, so future studies should also consider comparing microbiomes along the gradient of dams.

Despite Maryland’s Northern Map Turtles being the same population genetically [122], future conservation efforts may have to consider turtles above and below the dam separately because of the differences in microbiota. However, several bacteria associated with poor habitat quality were highly prevalent in all or most of our samples. Because of the relationship between organismal health and the microbiome, whole population microbiome monitoring will be necessary to ensure the survival of Northern Map Turtles in Maryland. Regardless, these results may aid in potential head starting efforts by providing information about wild Northern Map Turtle microbiomes, which could act as a baseline for captive individuals and may increase survival when released [123].

The present study is the first to examine the microbiome in Northern Map Turtles, and address how freshwater turtle microbiomes are impacted by a large hydroelectric dam. Further research needs to be done to grasp the relationship between Northern Map Turtles and their microbes. This includes use of additional sequencing methods, considering the limitations in sequencing a hypervariable region of the 16 s gene. Likewise, more work needs to be done to understand the effects that long-term exposure to dam sediments has on the microbiomes of freshwater organisms. Understanding the influence that dams have on organismal health will encourage conservation efforts and help maintain the persistence of those ecosystems.

## Supplementary Information

Below is the link to the electronic supplementary material.

Supplementary Material 1

Supplementary Material 3

## Data Availability

Sequencing files used in this study have been deposited into the NCBI Sequence Read Archive (SRA) with the sequential accession numbers SAMN50669242-SAMN50669377. Code for this project can be found in the following repository: https://github.com/hamchristoph/MapTurtleMicrobiome.
