# Supplementary material for: Hydroelectric Dam May Impact the Microbiome of an Endangered Northern Map Turtle (Graptemys geographica) Population
Source: Curr Microbiol. 2026 Mar 28;83(5):264. doi: 10.1007/s00284-026-04846-w (PMC13032988; doi:10.1007/s00284-026-04846-w)
Supplement: Supplementary file 2 — Supplementary Material 3 [file 284_2026_4846_MOESM2_ESM.docx]

**Table S1.** Summary of cloacal (A) and oral (B) microbiome swabs collected from Northern Map Turtles in Maryland during May-June 2022.

| **Dam Relativity** | **Site** | **Cloacal Swabs** | **Female** | **Male** | **Sex undetermined** | **Adult** | **Juvenile** | **Hatchling** |
| --- | --- | --- | --- | --- | --- | --- | --- | --- |
| *Above* | Broad Creek | 7 | 0 | 4 | 3 | 5 | 2 | 0 |
|  | Conowingo Creek | 11 | 5 | 2 | 4 | 8 | 3 | 0 |
| *Below* | Nesting Beach | 5 | 5 | 0 | 0 | 5 | 0 | 0 |
|  | Port Deposit | 12 | 10 | 1 | 1 | 11 | 1 | 0 |
|  | **Total** | **35** | **20** | **7** | **8** | **29** | **6** | **0** |

**A)**

| **Dam Relativity** | **Site** | **Oral Swabs** | **Female** | **Male** | **Sex undetermined** | **Adult** | **Juvenile** | **Hatchling** |
| --- | --- | --- | --- | --- | --- | --- | --- | --- |
| *Above* | Broad Creek | 6 | 0 | 3 | 3 | 4 | 2 | 0 |
|  | Conowingo Creek | 12 | 5 | 2 | 5 | 8 | 3 | 1 |
| *Below* | Nesting Beach. | 4 | 4 | 0 | 0 | 4 | 0 | 0 |
|  | Port Deposit | 11 | 8 | 1 | 2 | 9 | 1 | 1 |
|  | **Total** | **33** | **17** | **6** | **10** | **25** | **6** | **2** |

**B)**

**
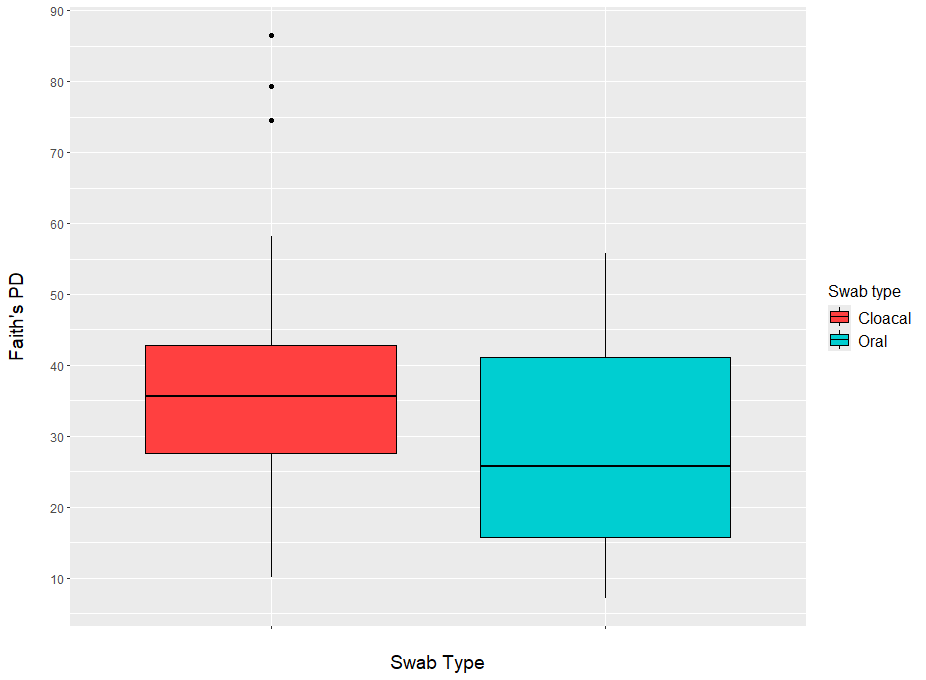
A)**

*

**
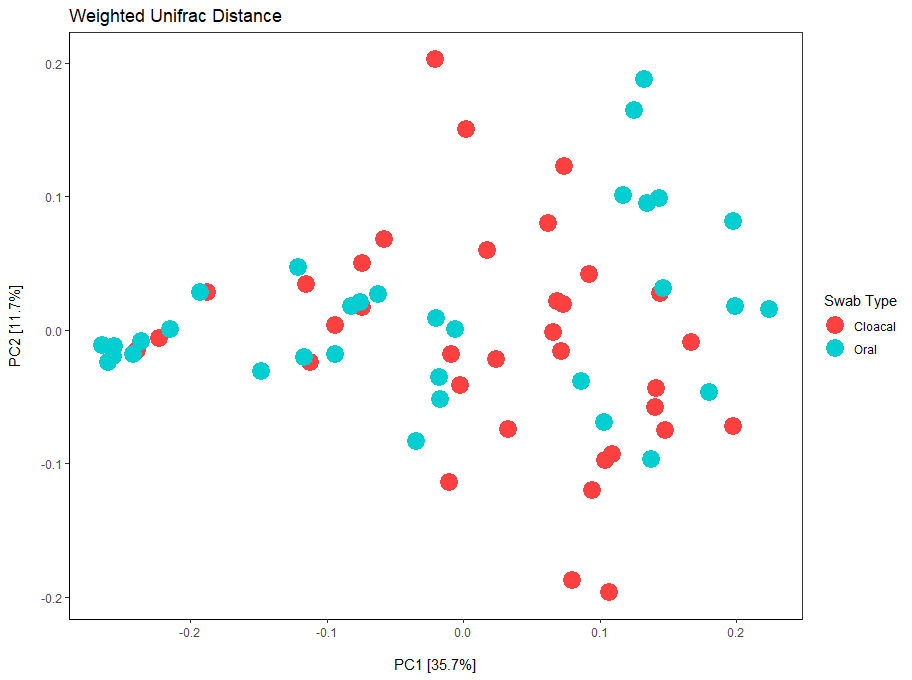
B)**

**Figure S1.** Phylogenetically weighted diversity metrics comparing cloacal and oral samples. **A)** Shows Faith’s PD, where Kruskal-Wallis showed significant statistical difference (*p=*0.022) **B)** Shows Weighted Unifrac, where PERMANOVA revealed statistical significance (*p* = 0.006).

**
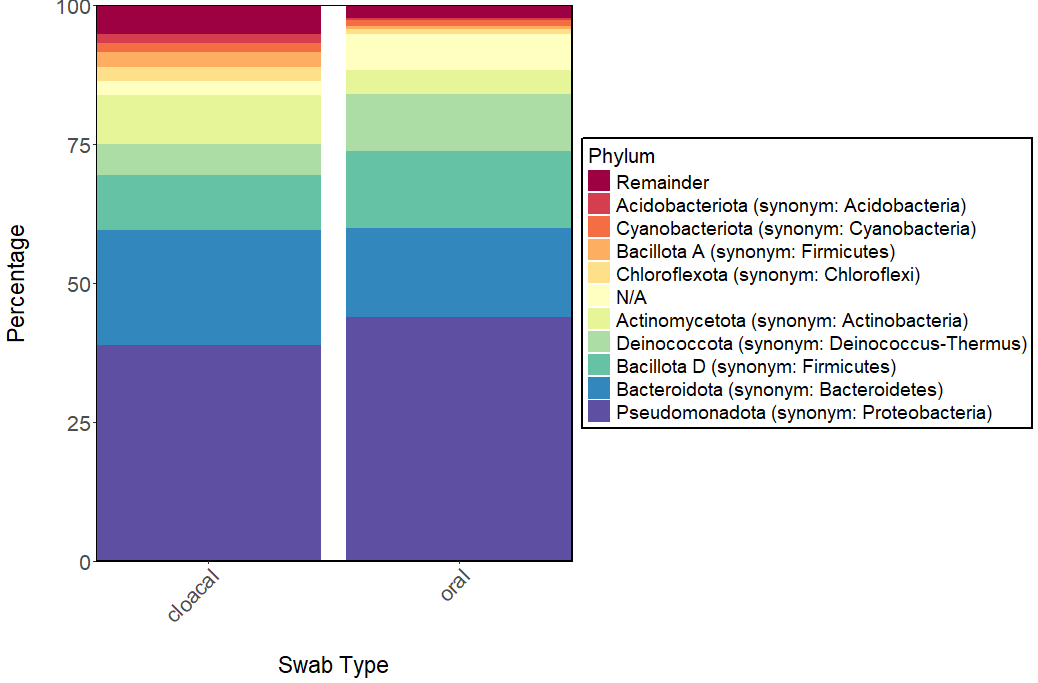
A)**

**
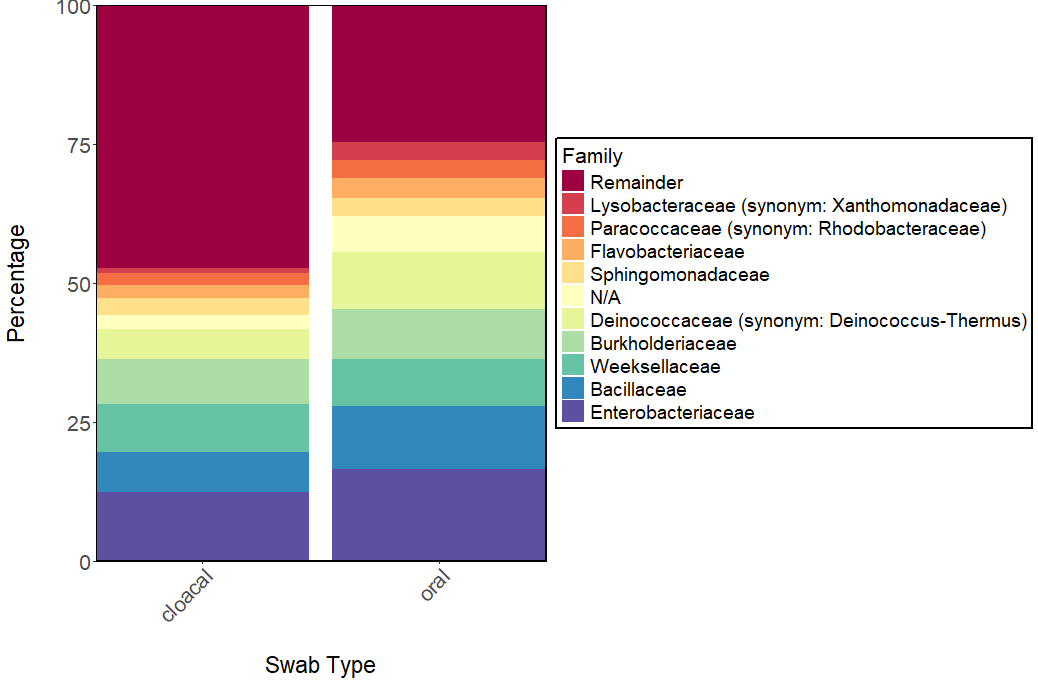
**

**B)**

**Figure S2.** Percentage of bacterial taxa for cloacal (n=35) and oral (n=33) swabs. A)

Represents the most common bacterial phyla, while B) represents the most common

bacterial families. “N/A” are the proportion of taxa that have not yet been identified at

that level. “Remainder” indicates the proportion of taxa that have been identified but are in

lower abundance.

**Table S2.** Summary of results from pairwise Kruskal-Wallis tests on Shannon Index and Faith’s Phylogenetic Diversity.

| **Swab Type** | **Variable** | **Comparison** | **Shannon Index** | | | **Faiths PD** | | |
| --- | --- | --- | --- | --- | --- | --- | --- | --- |
|  |  |  | H | *p-raw* | *p-adjusted* | H | *p-raw* | *p-adjusted* |
| *Cloacal* | Dam Relativity | Above vs. Below | 5.19 | **0.02*** | **0.02*** | 1.41 | 0.23 | 0.23 |
|  | Collection Site | Broad Creek vs. Conowingo Creek | 3.45 | 0.06 | 0.13 | 3.79 | **0.05*** | 0.15 |
|  |  | Broad Creek vs. Octoraro Creek | 0.32 | 0.57 | 0.73 | 0.06 | 0.81 | 0.81 |
|  |  | Broad Creek vs. Port Deposit | 0.26 | 0.61 | 0.73 | 0.11 | 0.74 | 0.81 |
|  |  | Conowingo Creek vs. Octoraro Creek | 4.39 | **0.04*** | 0.11 | 4.88 | **0.03*** | 0.15 |
|  |  | Conowingo Creek vs. Port Deposit | 6.37 | **0.01*** | 0.07 | 1.37 | 0.24 | 0.48 |
|  |  | Octoraro Creek vs. Port Deposit | 0.04 | 0.83 | 0.83 | 0.9 | 0.34 | 0.51 |
|  | Sex | Female vs. Male | 2.94 | 0.09 | 0.13 | 1.76 | 0.18 | 0.55 |
|  |  | Female vs. Undetermined | 0.01 | 0.92 | 0.92 | 0.66 | 0.42 | 0.56 |
|  |  | Male vs. Undetermined | 3.43 | 0.06 | 0.13 | 0.33 | 0.56 | 0.56 |
|  | Age Class | Adult vs. Juvenile | 1.39 | 0.24 | 0.24 | 0.008 | 0.93 | 0.93 |
| *Oral* | Dam Relativity | Above vs. Below | 0.52 | 0.47 | 0.47 | 8.16 | **0.004*** | **0.004*** |
|  | Collection Site | Broad Creek vs. Conowingo Creek | 0.56 | 0.45 | 0.54 | 1.26 | 0.26 | 0.31 |
|  |  | Broad Creek vs. Octoraro Creek | 1.64 | 0.20 | 0.40 | 2.23 | 0.14 | 0.20 |
|  |  | Broad Creek vs. Port Deposit | 1.01 | 0.31 | 0.47 | 2.27 | 0.13 | 0.20 |
|  |  | Conowingo Creek vs. Octoraro Creek | 3.76 | **0.05*** | 0.16 | 4.25 | **0.04*** | 0.12 |
|  |  | Conowingo Creek vs. Port Deposit | 0.19 | 0.67 | 0.67 | 6.06 | **0.01*** | 0.08 |
|  |  | Octoraro Creek vs. Port Deposit | 4.93 | **0.03*** | 0.16 | 0.61 | 0.43 | 0.43 |
|  | Sex | Female vs. Male | 0.02 | 0.89 | 0.89 | 1.59 | 0.21 | 0.31 |
|  |  | Female vs. Undetermined | 4.88 | **0.03*** | 0.08 | 9.08 | **0.003*** | **0.008*** |
|  |  | Male vs. Undetermined | 1.69 | 0.19 | 0.29 | 0.95 | 0.33 | 0.33 |
|  | Age Class | Adult vs. Hatchling | 2.19 | 0.14 | 0.21 | 3.78 | **0.05*** | 0.09 |
|  |  | Adult vs. Juvenile | 2.72 | 0.10 | 0.21 | 3.61 | 0.06 | 0.09 |
|  |  | Hatchling vs. Juvenile | 0 | 1 | 1 | 0.44 | 0.50 | 0.50 |

**
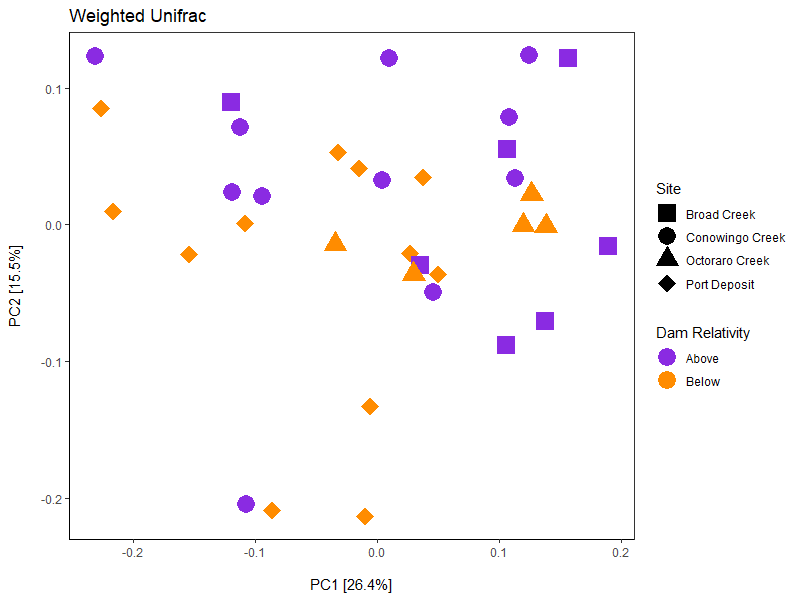
A)**


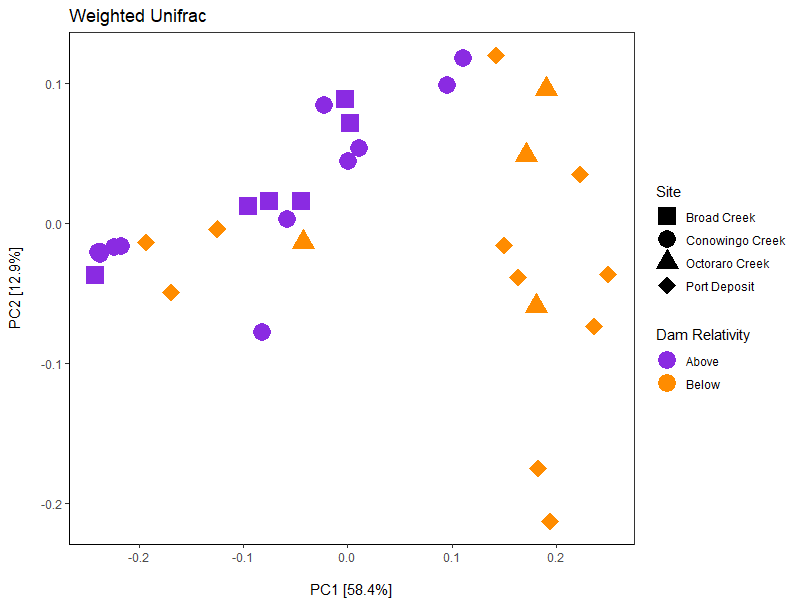
**B)**

**Figure S3.** Weighted Unifrac beta diversity ordination plots based on location. Samples collected above the dam are denoted by purple, while those collected below are denoted with orange. Individual sites are denoted by shape. **A)** Shows cloacal samples, which was significantly different by relativity to the dam (*p*=0.011) **B)** Shows oral samples, which was also significantly different by relativity to the dam (*p*=0.002).

**Table S3.** Summary of PERMANOVA and PERMDISP results for beta diversity metrics on both sample types. Statistically significant p-values (≤ 0.05) are denoted by an asterisk and bolded font.

| **Swab Type** | **Metric** | **Variable** | **Number of Groups** | **PERMANOVA** | | **PERMDISP** | |
| --- | --- | --- | --- | --- | --- | --- | --- |
|  |  |  |  | F | *p* | F | *p* |
| *Cloacal* | *Bray-Curtis* | Above/Below dam | 2 | 1.99 | **0.006*** | 2.59 | 0.107 |
|  |  | Collection Site | 4 | 1.69 | **0.001*** | 5.84 | **0.014*** |
|  |  | Sex | 3 | 1.50 | **0.003*** | 0.07 | 0.949 |
|  |  | Age Class | 2 | 1.60 | **0.018*** | 1.04 | 0.53 |
|  | *Weighted Unifrac* | Above/Below dam | 2 | 2.32 | **0.014*** | 4.90 | **0.034*** |
|  |  | Collection Site | 4 | 2.41 | **0.001*** | 3.62 | **0.023*** |
|  |  | Sex | 3 | 1.77 | **0.027*** | 2.09 | 0.174 |
|  |  | Age Class | 2 | 1.22 | 0.241 | 1.45 | 0.328 |
| *Oral* | *Bray-Curtis* | Above/Below dam | 2 | 6.87 | **0.001*** | 4.76 | **0.022*** |
|  |  | Collection Site | 4 | 3.32 | **0.001*** | 2.54 | 0.117 |
|  |  | Sex | 3 | 3.24 | **0.002*** | 2.28 | 0.144 |
|  |  | Age Class | 3 | 1.78 | **0.022*** | 26.7 | **0.005*** |
|  | *Weighted Unifrac* | Above/Below dam | 2 | 9.06 | **0.002*** | 2.47 | 0.085 |
|  |  | Collection Site | 4 | 3.27 | **0.008*** | 2.22 | 0.109 |
|  |  | Sex | 3 | 6.71 | **0.001*** | 0.25 | 1.27 |
|  |  | Age Class | 3 | 2.97 | **0.007*** | 9.64 | **0.011*** |

1. **
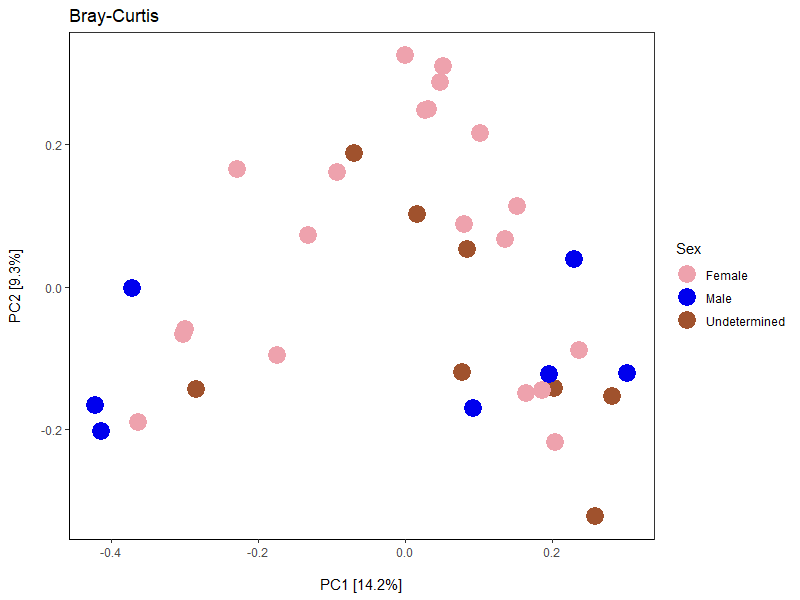
**

**
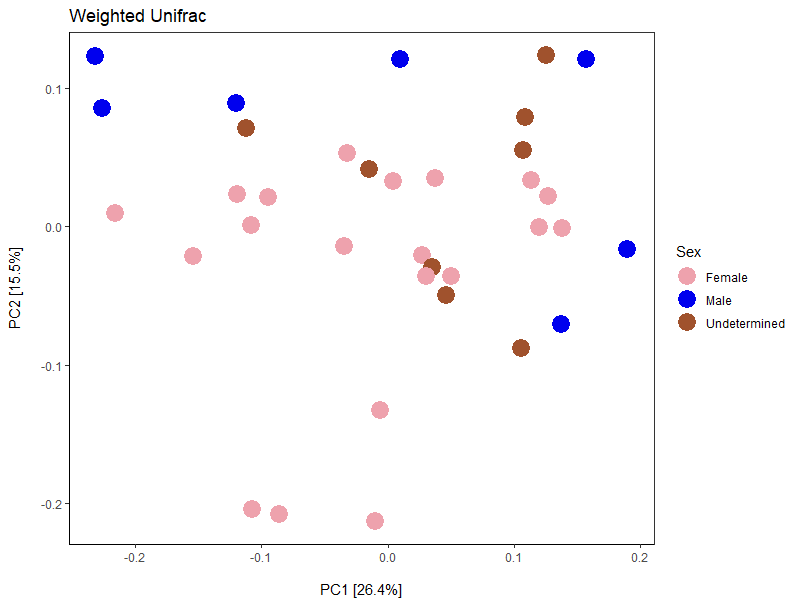
B)**

**Figure S4.** Beta diversity ordination plots for cloacal samples based on sex. Females (n=20) are denoted by pink, males (n=7) by blue, and undetermined (n=8) by brown. **A)** Shows Bray-Curtis dissimilarity, which was significant by sex (*p* = 0.03) **B)** Shows weighted unifrac, which was significant by sex (*p*=0.03).

**
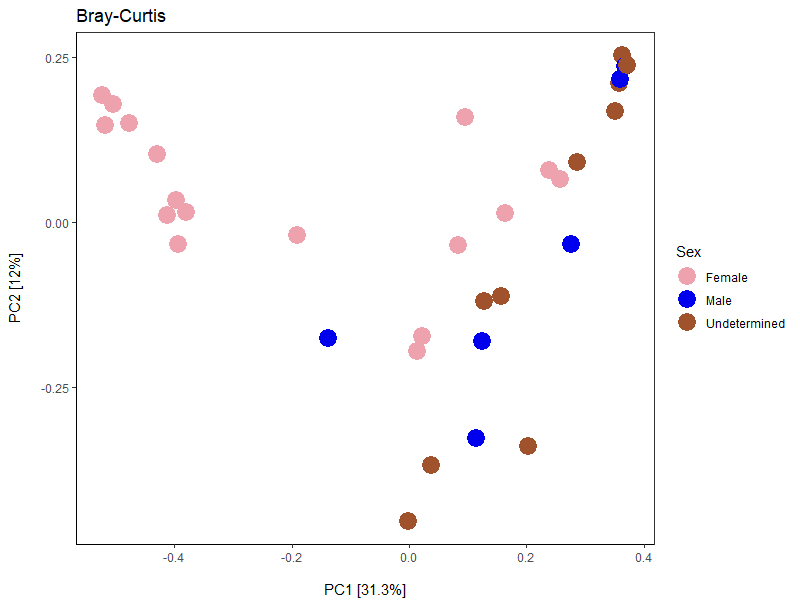
A)**

**
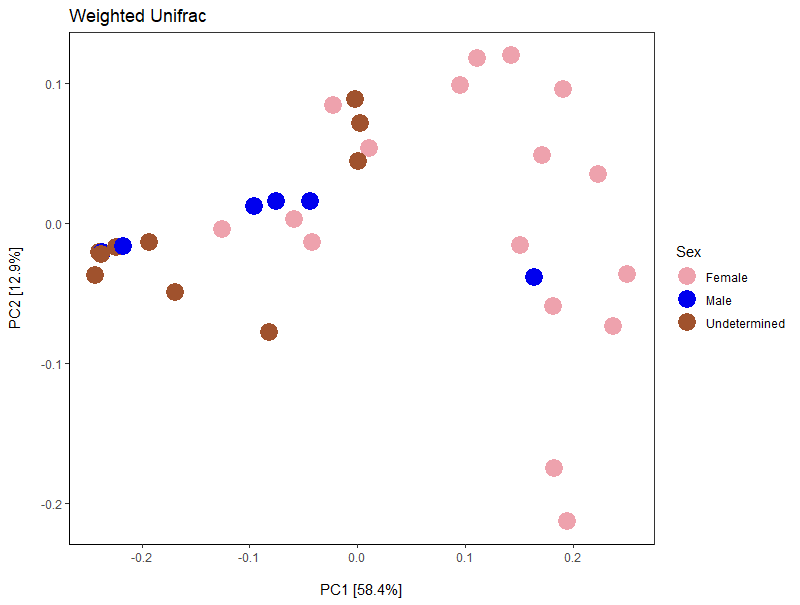
B)**

**Figure** **S5.** Beta diversity ordination plots for oral samples based on sex. Females (n=17) are denoted by pink, males (n=6) by blue, and undetermined (n=10) by brown **A)** Shows Bray-Curtis dissimilarity, which was significant by sex (*p* = 0.002) **B)** Shows weighted unifrac, which was significant by sex (*p*=0.001).

**
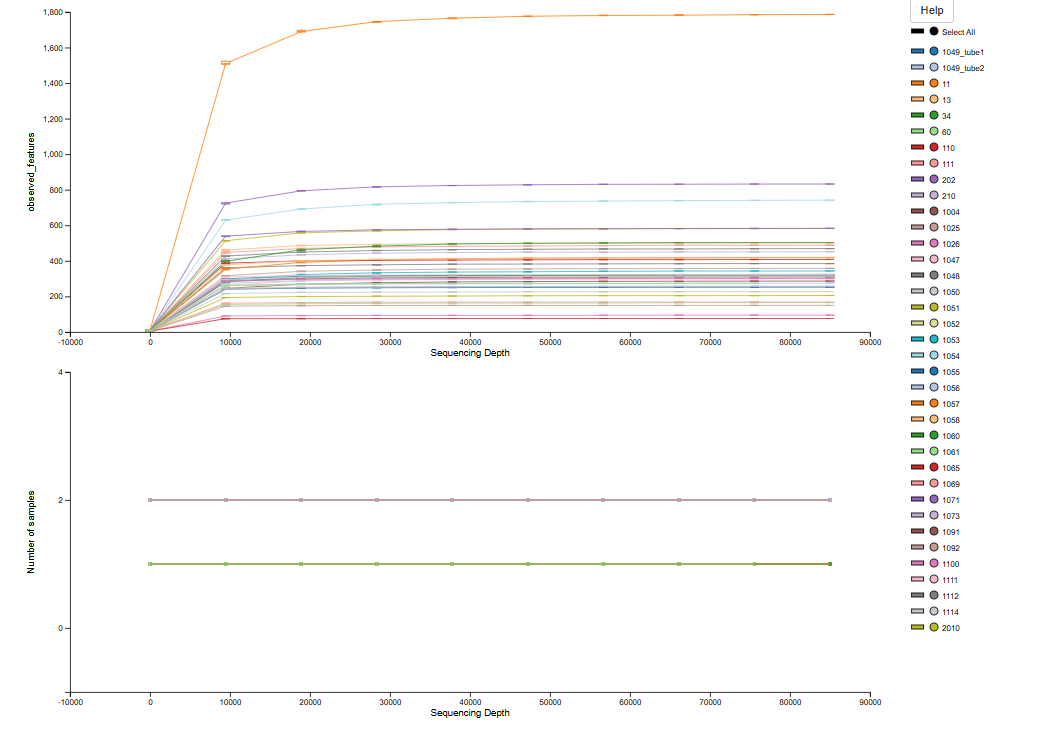
Figure S6.** Rarefaction curve for all samples. Sequences for this study were rarified at a depth of 80000.
